# Development of a non‐pharmacologic delirium management bundle in paediatric intensive care units

**DOI:** 10.1111/nicc.12809

**Published:** 2022-06-21

**Authors:** Rikke Louise Stenkjaer, Suzanne Forsyth Herling, Ingrid Egerod, Janne Weis, Monique van Dijk, Sapna Ravi Kudchadkar, Anne‐Sylvie Ramelet, Erwin Ista

**Affiliations:** ^1^ Department of Neonatology Copenhagen University Hospital Rigshospitalet Copenhagen Denmark; ^2^ The Neuroscience Centre Copenhagen University Hospital Rigshospitalet Copenhagen Denmark; ^3^ Department of Intensive Care Copenhagen University Hospital Rigshospitalet Copenhagen Denmark; ^4^ Department of Clinical Medicine University of Copenhagen Copenhagen Denmark; ^5^ Department of Pediatric Surgery Pediatric Intensive care, Erasmus MC – Sophia Children's Hospital Rotterdam the Netherlands; ^6^ Anesthesiology & Critical Care Medicine, Pediatrics, and Physical Medicine & Rehabilitation, Associate Vice Chair for Research, ACCM Johns Hopkins University School of Medicine, Charlotte Bloomberg Children's Center Baltimore Maryland USA; ^7^ Institute of Higher Education and Research in Healthcare (IUFRS) University of Lausanne Lausanne Switzerland; ^8^ Department Woman‐Mother‐Child Lausanne University Hospital Lausanne Switzerland

**Keywords:** delirium, delphi study, management, non‐pharmacological, paediatric intensive care unit

## Abstract

**Background:**

Non‐pharmacologic interventions might be effective to reduce the incidence of delirium in pediatric intensive care units (PICU).

**Aim:**

To explore expert opinions and generate informed consensus decisions regarding the content of a non‐pharmacologic delirium bundle to manage delirium in PICU patients.

**Study design:**

A two‐round online Delphi study was conducted from February to April 2021. PICU experts (nurses, physicians, researchers, physical therapists, play specialists, and occupational therapists) located in Europe, North America, South America, Asia, and Australia participated.

**Results:**

We developed a questionnaire based on the outcomes of a comprehensive literature search in the domains: 1) cognition support; 2) sleep support; and 3) physical activity support. Under these domains, we listed 11 strategies to promote support with 61 interventions. Participants rated the feasibility of each intervention on a 9‐point Likert scale (ranging from 1 strongly disagree to 9 strongly agree). A disagreement index and panel median were calculated to determine the level of agreement among experts. In the second round, participants reassessed the revised statements and ranked the interventions in each domain in order of importance for age groups: 0–2, 3–5, and 6–18 years of age. During the first Delphi round, 53 of 74 (72%) questionnaires were completed, and in the second round 45 of 74 (61%) were completed. Five of the highest ranked interventions across the age groups were: 1) developing a daily routine, 2) adjusting light exposure according to the time of day, 3) scheduling time for sleep, 4) providing eyeglasses and hearing aids if appropriate, 5) encouraging parental presence.

**Conclusions:**

Based on expert consensus, we developed an age‐specific non‐pharmacologic delirium bundle of interventions to manage delirium in PICU patients.

**Relevance to Clinical Practice:**

An age‐specific Non‐Pharmacological Delirium bundle is now ready to be tested in the PICU and will hopefully reduce pediatric delirium.

**What is known about the topic**

- Critically ill children admitted to the paediatric intensive care unit risk experiencing discomfort, pain, distress, withdrawal syndrome, and delirium.
- Non‐pharmacological multi‐component bundles for preventing delirium are effective in the adult intensive care unit settings.
- The Delphi method is a systematic, interactive method building on the opinions of a panel of experts within the field of a specific topic. After a number of rounds with questionnaires, consensus on the topic is reached.

**What this paper adds**

- Consensus of global experts on an age‐specific non‐pharmacological bundle to manage delirium in paediatric intensive care unit patients is presented.
- A parental and nurse‐driven non‐pharmacological delirium management bundle consisting of 1) developing a daily routine, 2) adjusting light exposure according to the time of day, 3) scheduling time for sleep, 4) providing eyeglasses and hearing aids if appropriate, 5) encouraging parental presence.
- The first step in development and evaluation of a complex intervention to manage paediatric delirium in the PICU is taken.

## 1 BACKGROUND

Critically ill children are likely to experience discomfort, pain, distress, withdrawal syndrome, and delirium in the paediatric intensive care unit (PICU). They often undergo unpleasant procedures such as insertion of intravenous lines, drains, catheters, suctioning when on mechanical ventilation, and other necessary, often recurrent, treatment procedures. Noise and light in the PICU environment add to the stressors experienced by critically ill children.^1^, ^2^ To reduce their suffering, healthcare providers regularly administer sedatives and analgesics to children, most commonly opioids and benzodiazepines.^3^ At the same time, extensive use of benzodiazepines and opioids carries the risk of prolonged mechanical ventilation, prolonged PICU stay, iatrogenic withdrawal syndrome (IWS), paediatric delirium (PD), and delusional memories.^4^, ^5^, ^6^, ^7^ Sleep disturbance and PD are frequently treated with additional sedatives, which may lead to a vicious cycle that contributes to increased child morbidity and mortality.^8^ Balancing adequate sedation, while avoiding over‐ and undersedation, is a PICU challenge.^9^

Systematic reviews based on hospitalized adult non‐ICU patients conclude that multi‐component non‐pharmacologic interventions reduce the occurrence of delirium compared with usual care.^10^, ^11^ However, studies in the adult intensive care unit (ICU) patients show that non‐pharmacologic interventions might be efficacious in reducing the incidence and duration of delirium.^12^, ^13^, ^14^ In the PICU setting, non‐pharmacologic interventions seem promising.^1^, ^15^ A recent study described a significant reduction of PD after implementation of a non‐pharmacologic bundle in children under 5 years and children after surgery after congenital heart disease surgery.^15^ Therefore, we assume that non‐pharmacologic interventions, such as promoting orientation, day‐night rhythm, and avoiding overstimulation from noise and light, might reduce delirium in critically ill children as well while causing no harm.

The Medical Research Council defines an intervention as being complex if it contains several components.^16^ Moreover, the council provides guidelines for the development and evaluation of complex interventions.^16^ This framework has four key phases: development, feasibility and piloting, evaluation, and implementation.^16^ The present Delphi study represents the development phase. We used the term “management” for both prevention and treatment of delirium, as our assumption is that the same interventions will reduce the incidence, relieve symptoms, and shorten the duration of PD.

## 2 AIMS

We aimed to explore expert opinions and generate informed decisions regarding the content of a “Non‐pharmacologic Delirium management Bundle” in PICU patients (NDB‐PICU).

## 3 METHODS

We performed a two‐round online Delphi study, to achieve agreement on the content of the NDB‐PICU among a group of international and interprofessional PICU experts. The strength of a Delphi study is its ability to rapidly obtain consensus opinions from an expert panel based on the assumption that group opinion is more valid than individual opinions on an issue that has no collective comprehension.^17^ The online Delphi format allowed us to reach a dispersed and varied group of international experts without compromising anonymity. We expected anonymity would minimize dominating opinions during consensus formation. We used a modified Delphi method, replacing the standard first round presenting the initial open‐ended questions and focus group discussions^17^ with a comprehensive literature search of non‐pharmacologic interventions in both adult and pediatric ICU patients^17^, ^18^, ^19^ (see Table S1).

### 3.1 The questionnaire

Based on the literature search we devised a questionnaire divided into three domains: 1) cognition support, 2) sleep support, and 3) physical activity support. In the three domains, we identified 11 strategies (for example, to promote a structure for the day, reducing noise, and dimming lights). Finally, the strategies were broken down into 61 specific interventions that could be initiated always in accordance with the medical condition and the developmental stage of the child for example, provide bright light during the daytime (See supplementary material, Table S2). To provide an overview of important age‐specific items, we subdivided the “child” category in the ranking session into three age groups defined by the author group: 1) 0–2 years (preverbal, neonate to toddler), 2) 3–5 years (preschool age), and 3) 6–18 years (school age). The final questionnaire was tested by the research group to ensure that the wording was understandable, the questions were relevant, and checking that each item contained only one question, and the statements were mutually exclusive.

### 3.2 Participants

Purposive sampling was used based on predetermined criteria to recruit experts representing clinical PICU practice. In this Delphi study, an expert was a PICU nurse, physician, researcher, physical therapist, play specialist, or occupational therapist. The inclusion criteria were both knowledge and practical experience of paediatric delirium as well as capacity and willingness to contribute.^17^ Applying a snowball sampling method, the members of the research group sent e‐mails to known colleagues with content knowledge, describing the aim of the Delphi study and asking them to participate and nominate other experts.^17^ We sent initial e‐mails to colleagues from North America (*n* = 5), South America (*n* = 1), Europe (*n* = 19), Australia (*n* = 4), and Asia (*n* = 3) to identify potential participants. The snowball sampling method resulted in 74 potential participants. They were informed about the aim of the study and expected time investment. We planned to limit the study to two Delphi rounds to prevent a low response rate, and ultimately to reduce attrition bias.^20^ We aimed to include at least 20 participants, which has been recommended to produce stable results.^21^

### 3.3 Data collection

Data were collected from the beginning of February to the beginning of April 2021 in the online Software survey tool Welphi version 4.0.^22^ Each participant received a personal invitation by e‐mail with a link to the questionnaire. In each of the two rounds, the participants were sent an information letter explaining the aim and content of that specific round, a consent form, the estimated time investment, and a deadline for completion. To optimize the response rate, we sent the participants a maximum of three reminders per round. Responses were anonymous both to the panel and the research group. A high response rate was important for the content validity of the results.^18^

The participants were asked to rate the feasibility in daily practice of the interventions of the proposed NDB‐PICU on a 9 point Likert scale (ranging from “1 strongly disagree” to “9 strongly agree”), and to provide a rationale for their response and including suggestions for improving the description. In addition, they were invited to comment, in their own words, how the suggested interventions could be revised. In the second round, all 74 participants received a general summary of the first round that presented the overall group results of the feasibility rating. The participants were asked to reconsider or confirm their opinion based on this new information and rank the approved, modified, and newly added interventions that were not part of the first Delphi round for their importance for delirium management, with a rank of 1 being most important.

### 3.4 Ethical considerations

This study was approved by the medical research ethics committee of the Erasmus University Medical Center, Rotterdam, the Netherlands (EMC 2017–1068). Participants completed an electronic consent for every round in which they participated and were ensured anonymity. This study was conducted according to the principles of the Declaration of Helsinki and in accordance with the Dutch Medical Research Involving Human Subjects Act.^23^ As all data were anonymous, permission to store data was not required.

### 3.5 Data analysis

For each intervention, we calculated a median rating and a disagreement index (DI) to determine the level of agreement. Median ratings between 7 and 9 were defined as relevant, 4 to 6 as somewhat relevant, and 1 to 3 as irrelevant. To calculate the DI, we divided the interpercentile range (IPR) (IPR 0.3–0.7) into the IPR adjusted for symmetry (IPRAS)^24^ (See supplementary material, Table S3). The cutoff for DI was defined as <0.2, indicating agreement. Finally, all interventions were categorized by the combination of the median score and the disagreement index. We distinguished three categories: 1) Appropriate: panel median score of 7–9, with the agreement (DI <0.2); 2) Uncertain/neutral: panel median score of 4–6, or with disagreement (DI ≥0.2) regardless of the median; and 3) Inappropriate: panel median score of 1–3, without disagreement.

The participants' written comments on the interventions were analysed by two of the researchers. The comments from the participants about the interventions were rephrased into new statements and included in the final round. The participants' overall ranking of the importance for each intervention was calculated as follows: the number of participants who ranked the interventions as “1” multiplied by the total number of interventions, the number who ranked the intervention as “2” multiplied by the number of interventions −1 and so on. The values for all rankings were summed for each intervention. The highest‐ranked intervention for each strategy and age group was included in the final recommendation of the NDB‐PICU.

## 4 RESULTS

We enrolled 74 participants to participate in the Delphi study. Participants were predominately from Europe and North America (*n* = 50) while a few represented South America and Australia (*n* = 3), and none from Asia. During the first Delphi round, 53 of 74 (72%) of the questionnaires were completed, and during the second round 45 of 74 (61%) were completed. All invited professions were represented in both rounds, but most participants were nurses and physicians who typically had more than 11 years of PICU experience (Table 1).

**TABLE 1:** Participants' characteristics

Characteristic	Delphi round 1	Delphi round 2
	*N* = 74	*N* = 74
	*n* (%)	*n* (%)
Response rate	53 (72)	45 (61)
Female	48 (91)	40 (89)
Age, years		
25–40	18 (34)	11 (24)
41–55	26 (49)	25 (56)
56–70	9 (17)	9 (20)
Geographic area		
Europe	34 (64)	29 (64)
South America	2 (4)	3 (7)
North America	16 (30)	12 (27)
Australia	1 (2)	1 (2)
Profession		
Physician	17 (32)	16 (36)
Nurse	20 (38)	16 (36)
Researcher	5 (9.5)	5 (11)
Physical therapist	6 (11)	6 (13)
Play specialist/occupational therapist	5 (9.5)	2 (4)
Education level		
Graduate	23 (43)	18 (40)
Undergraduate	3 (6)	2 (4)
Doctorate	27 (51)	25 (56)
PICU experience in years		
1–10	15 (28.5)	15 (34)
11–20	23 (43)	13 (29)
21–30	15 (28.5)	17 (37)

Abbreviation: PICU, pediatric intensive care unit.

For sixty‐one interventions in round 1, agreement among the participants was indicated with DI <0.2. Ten interventions with DI ≥0.2 were excluded. Eleven interventions needed to be modified and clarified based on input, and two new interventions were added: “staff should keep identification badge visible” and “use signs on patient door/bed to communicate that the child is sleeping or it is nap time.” Forty interventions did not require any modification. All 53 interventions indicated high agreement in round 2. Six interventions were modified and clarified, no new interventions were added, and two interventions were collapsed into one based on expert feedback (Table 2).

**TABLE 2:** Panel median and disagreement index (DI) in rounds 1 and 2

Domain, and related interventions	Round 1 panel median (DI)^a^	Round 2 panel median (DI)^a^
Support cognition		
Strategies for orientation provided by the healthcare professionals		
Explain who you are when you approach the child	9 (0.00)	
Address child by name	9 (0.00)	
Speak calmly and clearly	9 (0.00)	
Explain your role	9 (0.13)	
Stimulate the child's orientation by asking or describing time and place	9 (0.13)	
Ensure that the child is provided with age‐appropriate information	9 (0.00)	
Use uniform information, registered in the child's file^b^	8 (0.22)	‐
Use simple and short information about the ward, hospital, reason for admission	9 (0.13)	‐
*Make a fitting story with the child's own words about the ward, hospital, and reason for admission so that everybody uses the same language that the family does. Keep the story in the present*	‐	8 (0.13)
Ask what the child already knows about the course of disease and day plan^b^	8 (0.29)	‐
Explain plans for the day/evening/night	9 (0.13)	
Encourage consistency of staff caring for the child	9 (0.13)	‐
*Encourage consistent approach from staff caring for the child and consistent family support bedside for the child*	‐	9 (0.00)
Use a board to write the names of the staff assigned to the child	9 (0.19)	‐
*Use a board to write the names of staff on the shift who will care for the child and a little information about the child – what he likes to do and his interests*	‐	9 (0.13)
*Staff should keep identification badge visible*	‐	9 (0.13)
Strategies to promote a structure for the day		
Develop a day structure in collaboration with parents	9 (0.13)	
Use a board within the child's field of vision to show the structure of the day^b^	8 (0.49)	‐
Use a board to show the structure of the day using key words and pictograms^b^	8 (0.37)	‐
Provide bright light during the daytime	9 (0.13)	‐
*Provide bright light during the daytime. Open blinds at daytime – no bright light at nap times*		9 (0.00)
Strategies for improving the child's environment		
Provide appropriate lighting according to the time of day	9 (0.13)	
Provide a clock and calendar within the child's field of vision	9 (0.13)	
Orient the child's bed to support the circadian rhythm^b^	8 (0.29)	‐
Provide single room for each child	9 (0.13)	
Strategies for improving visual or hearing impairment		
Ensure that the child uses eyeglasses if appropriate whenever awake and ensure that the glasses are clean.	9 (0.00)	
Ensure that the child uses a hearing aid if appropriate when awake and ensure batteries are working	9 (0.00)	
Strategies to increase presence of parents		
Encourage the parents to be present	9 (0.00)	
Encourage the parents to take part in the daily activities	9 (0.00)	
Encourage visits from grandparents and friends if appropriate	8 (0.19)	
Provide parents with written information explaining the importance of being present and involved^b^	8 (0.29)	‐
*Provide parents with oral information explaining the importance of being present and involved without making them feel blamed if they are not able to be present 24/7*	‐	9 (0.13)
Strategies to improve the hominess of the child's surroundings		
*Encourage the child to do activities that they liked to do at home (e.g., watch television, use iPad)*		9 (0.00)
Encourage the child to watch preferred television programs and computer games in the daytime^b^	9 (0.29)	
Encourage presence of familiar objects around the bed	9 (0.00)	
Encourage the child to listen to preferred music	9 (0.13)	
Support sleep		
Strategies for bundling necessary nursing activities		
Schedule time for sleep.	9 (0.13)	
Bundle of necessary nursing activities	9 (0.13)	
Adjust the default hours for medication administration	8 (0.13)	
Adjust time for vital signs measurement	8 (0.19)	
Adjust the default hours for blood draws	9 (0.13)	
Strategies to promote homelike sleep rituals		
Play music according to the child's preferences	9 (0.13)	
Read aloud or tell a story to the child	9 (0.13)	
*Encourage the parents to read aloud or tell a story to the child*	‐	9 (0.00)
Provide sleep objects	9 (0.13)	
*Provide sleep objects such as teddy bear, sleeping pillow or cuddle cloth*	‐	9 (0.00)
Sing for or with the child	8 (0.13)	
*Encourage the parents to sing for or with the child*	‐	9 (0.13)
Strategies to reduce noise		
Close the door to the child's room and other rooms	9 (0.13)	‐
*Close the door if the staff is near to reduce noise*		9 (0.13)
Avoid loud talking in the child's room	9 (0.13)	
*Use signs on patient's door to communicate that the child is sleeping, or it is nap time*	‐	9 (0.00)
Provide colleagues with noise feedback	9 (0.13)	
In multiple‐bed settings, provide the child with headphones when listening to music or watching television	9 (0.13)	
Silence beepers and telephones if possible	9 (0.13)	
Provide earplugs for the child^b^	8 (0.37)	‐
Strategies to dim light		
Dim or turn off the artificial light around the child	9 (0.13)	
Dim light by using curtains or blinds	9 (0.13)	
Automatically turn off the light in adjacent rooms after a certain time	8 (0.29)	‐
Healthcare professionals should use a flashlight during the nightshift^b^	7 (0.42)	‐
Turn off tablets and smartphones before sleeping time	9 (0.13)	
Provide eye masks for the child^b^	7 (0.49)	‐
Dim monitor screen light and turn away from child	9 (0.13)	
Support physical activity		
Strategies for increasing mobilization		
Make activity goals visible in the child's room	9 (0.13)	
Encourage the child to be involved in activities of the day	9 (0.00)	
Provide physical therapy daily	9 (0.13)	‐
*Provide physical therapy when appropriate*		9 (0.00)
Document and evaluate daily mobilization goals	9 (0.13)	
Incorporate physical therapy in the activities of the day	9 (0.00)	
Document restrictions for mobilization	9 (0.13)	
Encourage parent involvement in mobilization activities	9 (0.00)	
Facilitate mobilization by removing or temporarily disconnect tubes and lines	9 (0.13)	
Document needs for assistive devices	9 (0.13)	

*Note*: Interventions in *italics* are modified or new interventions added based on the results of Delphi round 1; therefore, only the panel median (DI) of Delphi round 2 is available. The interventions that did not require modifications and were scored “feasible” in round 1 were accepted and not presented in round 2.

^a^ DI ≥0.2 indicates not feasible.

^b^ The interventions that were considered not feasible in round 1, with a DI ≥0.2, and therefore do not have a panel median (DI) in Delphi round 2.

After round 2, the expert group agreed on the feasibility of three domains: cognitive support, sleep support, and physical activity support, with 11 strategies covering 52 interventions (Table 2).

The highest‐ranked interventions for the 11 strategies and age groups, are listed in Table 3 and constitute an age‐appropriate NDB‐PICU. Five of the 11 interventions were similar among the age groups: 1) developing daily structure, 2) adjusting light exposure according to the time of day, 3) scheduling time for sleep, 4) providing eyeglasses and hearing aids if appropriate, 5) encouraging parents to be present. Furthermore, four interventions were shared by the 0–2 year age group and the 3–5 year age group, and two interventions were shared by the 3–5 year age group and the 6–18 year age group. The two younger age groups each had two specific interventions, and the 6–18 year age group had four (Table 3).

**TABLE 3:** Non‐pharmacologic delirium management program‐pediatric intensive care unit (NDB‐PICU) by age group^a^

Age group 0 to 2 years	Age group 3 to 5 years	Age group 6 to 18 years
Support cognition	Support cognition	Support cognition
Speak calmly and clearly		
	Explain who you are when you approach the child. Tell your name to the child	Explain who you are when you approach the child. Tell your name to the child
Develop a day structure in collaboration with parents		
Provide appropriate lighting according to the time of day		
Ensure that the child uses eyeglasses and hearing aids if appropriate when awake, and ensure that the glasses are clean, and batteries are working		
Encourage the parents to be present		
Encourage presence of familiar objects around the bed	Encourage presence of familiar objects around the bed	
		Encourage the child to do activities that they liked to do at home (e.g., watch television, use iPad)
*Support sleep*	*Support sleep*	*Support sleep*
Schedule time for sleep. Ask the parents about the usual sleep rhythm		
Provide sleep objects from home such as teddy bear, sleeping pillow, or cuddle cloth	Provide sleep objects from home such as teddy bear, sleeping pillow, or cuddle cloth	
		Play music according to the child's preferences. Consult parents
Avoid loud talking in the child's room	Avoid loud talking in the child's room	
		Close the door if the staff is near to reduce noise
Dim light by using curtains or blinds		
	Dim or turn off the artificial light around the child	Dim or turn off the artificial light around the child
*Support physical activity*	*Support physical activity*	*Support physical activity*
Document and evaluate daily mobilization goals	Document and evaluate daily mobilization goals	
		Make activity goals visible in the child's room

*Note*: Interventions highlighted with grey is similar between all age groups.

^a^ Interventions with the highest rank from all the strategies.

Overall, interventions with parent engagement were highly rated. Five interventions from each age group included parent collaboration, such as consulting parents about their child's daily structure; scheduling time for sleep; music preferences; bringing familiar objects from home such as comforting sleep objects; and overall encouragement of the parents to be present (see Tables S5, S6, and S7).

The participants responded that they found it difficult to rank the interventions because of the wide age span within the age groups, particularly in the oldest age group. Also, they commented that they found it difficult to rank equally important interventions (e.g., eyeglasses and hearing aids), and consequently these were combined. Furthermore, some participants found some interventions to be context‐specific, and although important, not feasible in their own setting (for example, one expert commented, “I agree with this opinion but quite often the structure and housing conditions of many PICUs do not allow for it”).

## 5 DISCUSSION

An international multidisciplinary panel of PICU experts reached consensus on the content of a non‐pharmacologic delirium management bundle (NDB‐PICU) for three different age groups using a Delphi method. We found that the five interventions general for all age groups preferred by PICU experts to prevent PD were: 1) developing a daily routine, 2) adjusting light exposure according to the time of the day, 3) scheduling time for sleep, 4) providing eyeglasses and hearing aids if appropriate, 5) encouraging parental presence.

The NDB‐PICU consists of three domains: cognitive support, sleep support, and physical activity support. Research in PICU concludes that hospitalization and critical illness have a negative impact on cognitive functions both in the short and long term.^25^, ^26^ Cognitive development relies on the presence of stimulating factors such as the presence of family or exposure to age‐appropriate educational activities.^25^ Interventions that support cognition account for more than half of the NDB‐PICU. To support these interventions, parents could play an important role in being present, playing, reading, and orienting the child. Encouraging family members to be present has been described previously and may decrease delirium rates.^27^, ^28^, ^29^ Interventions such as informing the families of delirium, displaying a daily schedule at the bedside, and letting parents bring familiar items from home such as blankets, pictures, and age‐appropriate toys have been positively evaluated.^27^, ^29^ Parents feel more engaged and relieved when they can care for their child through such interventions.^27^ Although parents might be stressed, we know from adult literature that it looks promising to have a family member delivering a non‐pharmacologic intervention to reduce delirium in critically ill adults.^30^

To promote sleep in the adult ICU, the use of eye masks and/or earplugs has been tested and significantly decreases the rate of delirium.^31^ For children, suggestions in a family‐centered toolkit have been made to provide eye masks, dim the light at night, and provide headphones to reduce noise.^27^ In our Delphi study, such interventions were rejected. Rather, interventions to modify the environment in the room by avoiding loud talk and dimming lights with curtains or blinds were prioritized. Experts may have rejected the idea of using eye masks and earplugs because such devices may be unfamiliar or disruptive for children, who, unlike adults, are unaccustomed to them. The children may become scared because of the sudden lack of sight and something in their eyes and try to remove it. Sleep intervention support needs to be attuned to the child's age and sleep preferences, while parents should recommend day‐night routines that reflect home routines, such as usual bedtime, objects to take to bed, such as cuddle toys, or listening to preferred music.

In the NDB‐PICU, strategies to increase mobilization were highly ranked among the participants. The intervention “provide physical therapy daily” was modified to “provide physical therapy as considered appropriate”, indicating that the participants found physical therapy to be important for most children, but that level and type should be based on developmental age and criticality. It has previously been established that PICU clinicians find early mobilization important.^32^ Despite this, mobilization does not occur in 25% of critically ill children across Europe, for instance, because of tubes or catheters.^33^ The most common activity seems to be children being held by family or nurse.^33^ Thus, the application of non‐pharmacologic delirium management interventions as defined in the NDB‐PICU is intertwined with other aspects such as early mobilization and family presence as described in the Assessing Pain, Both Spontaneous Awakening and Breathing Trials, Choice of Sedation, Delirium Monitoring/Management, Early Exercise/Mobility, and Family Engagement/Empowerment (ABCDEF) paediatric bundle.^34^

A non‐pharmacologic nursing bundle consisting of eight interventions during dayshift and four interventions during nightshift did not show significant delirium prevention.^35^ Similar to the NDB‐PICU interventions, that bundle^35^ included tenets of “encouraging family presence when possible,” “bringing familiar objects,” providing frequent orientation to the patient, providing glasses or hearing aids when needed, and controlling noise levels to promote sleep. The authors speculated that the bundle did not have the intended effect because of inadequate delirium assessment, a lack of knowledge about PD, and a lack of adherence to the bundle.^35^ Another non‐pharmacologic paediatric delirium bundle, developed as a checklist, led to significant noise reduction in the PICU.^1^ Building on these findings, we argue that the application of an age‐specific NDB‐PICU with an 11‐intervention checklist could manage delirium provided regular delirium screening, awareness of PD, and interprofessional involvement occur (See supplementary material, Tables S8, S9, and S10). Furthermore, we expect that nurses with a delirium management strategy are likely to feel more empowered to act upon a positive delirium assessment.^1^

This study allowed us to develop the NDB‐PICU, which needs to be tested for efficacy on delirium reduction in PICU patients. With step one of the MRC framework completed, the next phase of this complex intervention can be carried out,^16^ namely the feasibility, pilot testing, and evaluation.

### 5.1 Strengths and limitations

One strength of this study was the composition and size of the expert panel from different professions and countries worldwide. Another strength was the high response rate, which positively influenced the validity and robustness of the results. Nevertheless, the study has also had several limitations. Ironically, the high agreement among participants on most interventions was a limitation. It is likely that agreement was high because the questionnaire was positively formulated based on evidence‐based interventions from the literature. Another limitation was the recruitment procedure. We missed participants from Asia and Africa. We could have improved inclusion if we had recruited participants through international societies. Potential strengths and limitations were the chosen age groups. We created three age groups to clarify age‐specific interventions. The wide age span from 6 to 18 years is justified by the fact that from that age onwards language development is such that the children are able to express themselves well about what they like in their situation. Obviously, there are differences between children of different ages (e.g., type of preferred music) but others like a daily structure and parental presence are applicable for all age groups. Parents are around to fill in the gaps in our knowledge about the child. The fact that the COVID‐19 pandemic was occupying ICU personnel worldwide may also have reduced the time available to potential participants.

### 5.2 Conclusion and relevance to clinical practice

In this Delphi study, international and interprofessional PICU experts identified non‐pharmacologic delirium interventions consisting of developing a daily routine, adjusting light exposure according to the time of day, scheduling time for sleep, providing eyeglasses and hearing aids if appropriate, and encouraging parental presence. Nurse‐driven interventions with parental engagement are preferred in the management of delirium in PICU patients. The next step will be feasibility testing and piloting of the NDB‐PICU investigating parental engagement in clinical practice, evaluation, and implementation of the bundle.

## AUTHOR CONTRIBUTIONS

**Rikke Louise Stenkjaer**: Conceived and designed the analysis, Collected the data, Contributed data or analysis tools, Performed the analysis, Wrote the paper; **Suzanne Forsyth Herling**: Conceived and designed the analysis, Contributed data or analysis tools, Performed the analysis, Wrote the paper: **Ingrid Egerod**: Conceived and designed the analysis, Contributed data or analysis tools, Wrote the paper; **Janne Weis**: Conceived and designed the analysis, Contributed data or analysis tools, Wrote the paper; **Monique van Dijk**: Conceived and designed the analysis, Contributed data or analysis tools, Wrote the paper; **Sapna Kudchadkar**: Conceived and designed the analysis, Contributed data or analysis tools, Wrote the paper; **Anne‐Sylvie Ramelet**: Conceived and designed the analysis, Contributed data or analysis tools, Wrote the paper; **Erwin Ista**: Conceived and designed the analysis, Contributed data or analysis tools, Performed the analysis, Wrote the paper.

## FUNDING INFORMATION

Department of Neonatology, Copenhagen University Hospital Rigshospitalet, Blegdamsvej 9, Copenhagen 2100, Denmark. The Novo Nordisk Foundation, Denmark (NNF 20OC0066074). Department of Pediatric Surgery, Pediatric Intensive care, Erasmus MC – Sophia Children's Hospital, Rotterdam, the Netherlands.

## Supporting information


**Table S1.** Search string.
**Table S2.** The questionnaire in round 1.
**Table S3.** Calculating level of agreement.
**Table S4.** The final questionnaire*.
**Table S5.** Ranking of the interventions for children from 0 to 2 years of age.
**Table S6.** Ranking of the interventions for children from 3 to 5 years of age.
**Table S7.** Ranking of the interventions for children from 6 to 18 years of age.
**Table S8.** Non‐pharmacologic Delirium management Program (NDB‐PICU) – checklist for children from 0 to 2 years of age.
**Table S9.** Non‐pharmacologic Delirium management Program (NDB‐PICU)—checklist for children from 3 to 5 years of age.
**Table S10.** Non‐pharmacologic Delirium management Program (NDB‐PICU)—checklist for children from 6 to 18 years of age.
