# Supplementary material for: Development of a non‐pharmacologic delirium management bundle in paediatric intensive care units
Source: Nurs Crit Care. 2022 Jun 21;27(6):867–76. doi: 10.1111/nicc.12809 (PMC10084175; doi:10.1111/nicc.12809)
Supplement: Supplementary file 1 — Table S1. Search string. Table S2. The questionnaire in round 1. Table S3. Calculating level of agreement. Table S4. The final questionnaire*. Table S5. Ranking of the interventions for children from 0 to 2 years of age. Table S6. Ranking of the interventions for children from 3 to 5 years of age. Table S7. Ranking of the interventions for children from 6 to 18 years of age. Table S8. Non‐pharmacologic Delirium management Program (NDB‐PICU) – checklist for children from 0 to 2 years of age. Table S9. Non‐pharmacologic Delirium management Program (NDB‐PICU)—checklist for children from 3 to 5 years of age. Table S10. Non‐pharmacologic Delirium management Program (NDB‐PICU)—checklist for children from 6 to 18 years of age. [file NICC-27-867-s001.docx]

**Supplementary material**

[Table A: Search string 2](#_Toc85211510)

[Table B: The questionnaire in round 1 3](#_Toc85211511)

[Table C: Calculating level of agreement 6](#_Toc85211512)

[Table D: The final questionnaire^*^ 7](#_Toc85211513)

[Table E: Ranking of the interventions for children from 0 to 2 years of age 9](#_Toc85211514)

[Table F: Ranking of the interventions for children from 3 to 5 years of age 11](#_Toc85211515)

[Table G: Ranking of the interventions for children from 6 to 18 years of age 13](#_Toc85211516)

[Table H: Non-pharmacologic Delirium management Program (NDB-PICU) – checklist for children from 0 to 2 years of age 15](#_Toc85211517)

[Table I: Non-pharmacologic Delirium management Program (NDB-PICU) – checklist for children from 3 to 5 years of age 16](#_Toc85211518)

[Table J: Non-pharmacologic Delirium management Program (NDB-PICU) – checklist for children from 6 to 18 years of age 17](#_Toc85211519)

# Table A: Search string

((("delirium"[MeSH Terms] OR "delirium"[All Fields]) OR "deliriums"[All Fields]) AND ((((("paediatrics"[All Fields] OR "pediatrics"[MeSH Terms]) OR "pediatrics"[All Fields]) OR "paediatric"[All Fields]) OR "pediatric"[All Fields]) OR (((((("child"[MeSH Terms] OR "child"[All Fields]) OR "children"[All Fields]) OR "child s"[All Fields]) OR "children s"[All Fields]) OR "childrens"[All Fields]) OR "childs"[All Fields]))) AND (((("intensive care units"[MeSH Terms] OR (("intensive"[All Fields] AND "care"[All Fields]) AND "units"[All Fields])) OR "intensive care units"[All Fields]) OR (("critical illness"[MeSH Terms] OR ("critical"[All Fields] AND "illness"[All Fields])) OR "critical illness"[All Fields])) OR (("critical"[All Fields] OR "critically"[All Fields]) AND "ill"[All Fields]))

# Table B: The questionnaire in round 1

**Support cognition**

1. Strategies for orientation provided by the healthcare professionals

- Explain who you are when you approach the child. *Tell your name to the child*
- Speak calmly and clearly
- Explain your role. *What are you going to do together with the child in your shift?*
- Address child by name
- Use uniform information, registered in the child’s file. *Does the child like to be touched by hand while you speak to him or her?*
- Stimulate the child’s orientation by asking or describing time and place. *Tell which weekday and date it is and where the child is*
- Use simple and short information about the ward, hospital, reason for admission
- Ask what the child already knows about the course of disease and day plan
- Explain plans for the day/evening/night*. E.g.* *should there be any examinations during the shift?*
- Ensure that the child is provided with age-appropriate information
- Encourage consistency of staff caring for the child
- Use a board to write the names of the staff assigned to the child. *Write the names of the nurse and the physicians during each shift*

1. Strategies to promote a structure for the day
   - Develop a day structure in collaboration with parents *What is the child’s normal day structure? When does the child goes to sleep? When does the child wake up in the morning?*

- Use a board within the child’s field of vision to show the structure of the day. *The structure of the day should include activities such as mobilization, sleep, visiting hours*
- Use a board to show the structure of the day using key words and pictograms
- Provide bright light during the daytime. *Draw curtains so that the daylight can come in and turn on the light*

1. Strategies for improving the child’s environment
   - Provide a clock and calendar within the child’s field of vision

- Provide appropriate lighting according to the time of day
- Orient the child’s bed to support the circadian rhythm. *Orient the child’s bed to perceive daylight/darkness from a window*
- Provide single room for each child

1. Strategies for improving visual or hearing impairment

- Ensure that the child uses eyeglasses if appropriate whenever awake and ensure that the glasses are clean. *Ask the parents when the child uses their eyeglasses during the daytime.*
- Ensure that the child uses a hearing aid if appropriate when awake and ensure batteries are working

1. Strategies to increase presence of parents

- Encourage the parents to be present
- Encourage the parents to take part in the daily activities
- Encourage visits from grandparents and friends if appropriate
- Provide parents with written information explaining the importance of being present and involved

1. Strategies to improve the hominess of the child’s surroundings

- Encourage presence of familiar objects around the bed. C*hild’s own pillow, pictures of family, friends, pets, posters, drawings*
- Encourage the child to watch preferred television programs and computer games in the daytime. *Consult parents*
- Encourage the child to listen to preferred music. *Consult parents*

**Support sleep**

1. Strategies for bundling necessary nursing activities
   - Schedule time for sleep. *Ask the parents about the usual sleep rhythm.*
   - Adjust of the default hours for medication administration
   - Adjust the default hours for blood draws. *Do routine blood draws when the child is awake.*
   - Adjust time for vital signs measurement. *Do routine measurement when the child is awake.*
   - Bundle of necessary nursing activities. *Conduct suctioning or diaper change when the child is awake if possible.*
2. Strategies to promote homelike sleep rituals
   - Play music according to the child’s preferences. *Consult parents*
   - Read aloud or tell a story to the child. *E.g. by parents, nurse or audio book*
   - Sing for or with the child. *Parents or nurse*
   - Provide sleep objects. *Sleeping pillow, teddy bear or cuddle cloth.*
3. Strategies to reduce noise
   - Close the door to the child’s room and other rooms. *Staff room or utility room.*
   - Avoid loud talking in the child’s room. *Healthcare professionals should talk quietly.*
   - Provide colleagues with noise feedback
   - Silence beepers and telephones if possible
   - In multiple-bed settings, provide the child with headphones when listening to music or watching television
   - Decrease alarm volumes and increase alarm boundaries for the monitor or ventilator
   - Provide earplugs for the child
4. Strategies to dim light
   - Dim light by using curtains or blinds
   - Dim or turn off the artificial light around the child
   - Automatically turn off the light in adjacent rooms after a certain time
   - Healthcare professionals should use a flashlight during the nightshift
   - Dim monitor screen light and turn away from child
   - Provide eye masks for the child
   - Turn off tablets and smartphones before sleeping time

**Support physical activity**

Strategies for increasing mobilization

- Document restrictions for mobilization
- Document and evaluate daily mobilization goals
- Make activity goals visible in the child’s room
- Document needs for assistive devices. *Crutches, braces, wheelchair*
- Provide physical therapy daily
- Incorporate physical therapy in the activities of the day
- Facilitate mobilization by removing or temporarily disconnect tubes and lines
- Encourage parent involvement in mobilization activities
- Encourage the child to be involved in activities of the day. *E.g. encourage the child to participate in personal hygiene*

# Table C: Calculating level of agreement

The intervention “Use a board within the child’s field of vision to show the structure of the day” is used as an example

| ROUND 1  Domaine: Support cognition  Strategies: Strategies to promote a structure for the day | Use a board within the child’s field of vision to show the structure of the day | |
| --- | --- | --- |
| Frequency table  Respondents n=53 | Score | Frequency |
|  | 1 | 1 |
|  | 2 | 0 |
|  | 3 | 3 |
|  | 4 | 0 |
|  | 5 | 6 |
|  | 6 | 7 |
|  | 7 | 3 |
|  | 8 | 7 |
|  | 9 | 26 |
| 50^th^ percentile (median) | 8.00 | |
| 30^th^ percentile | 6.00 | |
| 70^th^ percentile | 9.00 | |
| Interpercentile range (IPR) | IPR = (70^th^ percentile – 30^th^ percentile)  IPR = 9.00 – 6.00 = 3.00 | |
| Interpercentile range central point (IPRCP) | IPRCP= (70^th^ percentile + 30^th^ percentile)/2  IPRCP= (9.00 + 6.00)/2= 7.50 | |
| Asymmetry index (AI) | AI = \|5 – IPRCP\|  AI = \|5 – 7.50\| = 2.50 | |
| Interpercentile range adjusted for symmetry (IPRAS) | IPRAS = 2.35 + (AI x 1.5)  IPRAS = 2.35 + (2.50 x 1.5) = 6.10 | |
| Disagreement index (DI) | DI = IPR/IPRAS  DI = 3.00/6.10 = 0.49 | |
| Interpretation | DI = 0.49 (< 1)  DI < 1 indicates agreement | |

# Table D: The final questionnaire^*^

**Support cognition**

1. Strategies for orientation provided by the healthcare professionals

- Explain who you are when you approach the child. *Tell your name to the child.*
- Speak calmly and clearly
- Explain your role. *What are you going to do together with the child in your shift?*
- Address child by name
- Stimulate the child’s orientation by asking or describing time and place. *Tell which weekday and date it is and where the child is*

***Explain using the child’s own words about the ward, hospital and reason for admission, so that everybody around the child uses the same terminologies***

- Explain plans for the day/evening/night*. E.g.* *should there be any examinations during the shift?*
- Ensure that the child is provided with age-appropriate information
- ***Encourage consistent non-pharmacologic approach from staff caring for the child and consistent family support bedside for the child***
- Use a board to write staff the names of staff on the shift who will care for the child and a little information about the child – what he likes to do and his interests
- ***Staff should keep identification badge visible***

1. Strategies to promote a structure for the day

- Develop a day structure in collaboration with parents *What is the child’s normal day structure? When does the child goes to sleep? When does the child wake up in the morning?*
- Provide bright light during the daytime. Open blinds at daytime – no bright light at nap times.

1. Strategies for improving the child’s environment

- Provide a clock and calendar within the child’s field of vision
- Provide appropriate lighting according to the time of day
- Provide single room for each child

1. Strategies for improving visual or hearing impairment

- ***Ensure that the child uses eyeglasses and hearing aids if appropriate when awake and ensure that the glasses are clean, and batteries are working***

1. Strategies to increase presence of parents

- Encourage the parents to be present
- Encourage the parents to take part in the daily activities
- Encourage visits from grandparents and friends if appropriate
- ***Provide parents with oral information explaining the importance of being present if they are able to be present 24/7***

1. Strategies to improve the hominess of the child’s surroundings

- Encourage presence of familiar objects around the bed. C*hild’s own pillow, pictures of family, friends, pets, posters, drawings.*
- Encourage the child to do activities that they liked to do at home (e.g., watch television, use iPad). Consult parents
- Encourage the child to listen to preferred music. *Consult parents*

**Support sleep**

1. Strategies for bundling necessary nursing activities
   - Schedule time for sleep. *Ask the parents about the usual sleep rhythm*
   - Adjust the default hours for medication administration
   - Adjust the default hours for blood draws. *Do routine blood draws when the child is awake*
   - Adjust time for vital signs measurement. *Do routine measurement when the child is awake*
   - Bundle of necessary nursing activities. *Conduct suctioning or diaper change when the child is awake if possible*
2. Strategies to promote homelike sleep rituals
   - Play music according to the child’s preferences. *Consult parents*
   - ***Encourage the parents provide age appropriate rituals individually tailored for the child as read aloud or tell a story or sing for and with to the child***
   - Encourage the parents to sing for or with the child
   - ***Provide sleep objects from home such as teddy bear, sleeping pillow or cuddle cloth***
3. Strategies to reduce noise
   - Close the door if the staff is near to reduce noise
   - ***Use signs on patient’s door/bed to communicate that the child is sleeping or it is nap time***
   - Avoid loud talking in the child’s room. *Healthcare professionals should talk quietly.*
   - Provide colleagues with noise feedback
   - Silence beepers and telephones if possible
   - In multiple-bed settings, provide the child with headphones when listening to music or watching television
4. Strategies to dim light
   - Dim light by using curtains or blinds
   - Dim or turn off the artificial light around the child
   - Dim monitor screen light and turn away from child
   - Turn off tablets and smartphones before sleeping time

**Support physical activity**

1. Strategies for increasing mobilization

- Document restrictions for mobilization
- Document and evaluate daily mobilization goals
- Make activity goals visible in the child’s room
- Document needs for assistive devices. *Crutches, braces, wheelchair*
- Provide physical therapy when appropriate
- Incorporate physical therapy in the activities of the day
- Facilitate mobilization by removing or temporarily disconnect tubes and lines
- Encourage parent involvement in mobilization activities
- Encourage the child to be involved in activities of the day. *E.g. encourage the child to participate in personal hygiene*

***^*^Italicized interventions are modified from round 2 or newly added***

# Table E: Ranking of the interventions for children from 0 to 2 years of age

| Risk factor  Ranking interventions for **children from 0 to 2 years of age** | Total score^*^ |
| --- | --- |
| **Support cognition** |  |
| **Strategies for orientation provided by the healthcare professionals** |  |
| Speak calmly and clearly | 443 |
| Address child by name | 436 |
| Explain who you are when you approach the child. *Tell your name to the child.* | 374 |
| Ensure that the child is provided with age-appropriate information | 291 |
| Explain your role. *What are you going to do together with the child in your shift?* | 290 |
| Encourage consistent approach from staff caring for the child and consistent family support bedside for the child | 288 |
| Make a fitting story with the child’s own words about the ward, hospital, and reason for admission, so that everybody uses the same language that the family does. Keep the story in the present | 248 |
| Stimulate the child’s orientation by asking or describing time and place. *Tell which weekday and date it is and where the child is.* | 198 |
| Explain plans for the day/evening/night*. E.g.* *should there be any examinations during the shift?* | 192 |
| Use a board to write the names of staff on the shift who will care for the child and a little information about the child – what he likes to do and his interests. | 138 |
| Staff should keep identification badge visible | 72 |
| **Strategies to promote a structure for the day** |  |
| Develop a day structure in collaboration with parents *What is the child’s normal day structure? When does the child goes to sleep? When does the child wake up in the morning?* | 69 |
| Provide bright light during the daytime*. Open blinds at daytime – no bright light at nap times.* | 66 |
| **Strategies for improving the child’s environment** |  |
| Provide appropriate lighting according to the time of day | 126 |
| Provide single room for each child | 90 |
| Provide a clock and calendar within the child’s field of vision | 54 |
| **Strategies for improving visual or hearing impairment** |  |
| Ensure that the child uses eyeglasses if appropriate whenever awake and ensure that the glasses are clean. *Ask the parents when the child uses their eyeglasses during the daytime.* | 75 |
| Ensure that the child uses a hearing aid if appropriate when awake and ensure batteries are working | 60 |
| **Strategies to increase presence of parents** |  |
| Encourage the parents to be present | 151 |
| Encourage the parents to take part in the daily activities | 129 |
| Provide parents with oral information explaining the importance of being present and involved without making them feel blamed if they are not able to be present 24/7 | 110 |
| Encourage visits from grandparents and friends if appropriate | 60 |
| **Strategies to improve the hominess of the child’s surroundings** |  |
| Encourage presence of familiar objects around the bed. C*hild’s own pillow, pictures of family, friends, pets, posters, drawings.* | 128 |
| Encourage the child to do activities that they liked to do at home (e.g., watch television, use iPad). Consult parents | 78 |
| Encourage the child to listen to preferred music. *Consult parents* | 64 |
| **Support sleep** |  |
| **Strategies for bundling necessary nursing activities** |  |
| Schedule time for sleep. *Ask the parents about the usual sleep rhythm* | 202 |
| Bundle necessary nursing activities. *Conduct suctioning or diaper change when the child is awake if possible* | 149 |
| Adjust the default hours for medication administration | 118 |
| Adjust the default hours for blood draws. *Do routine blood draws when the child is awake* | 105 |
| Adjustment time for vital signs measurement. *Do routine measurement when the child is awake* | 101 |
| **Strategies to promote homelike sleep rituals** |  |
| Provide sleep objects such as teddy bear, sleeping pillow or cuddle cloth | 150 |
| Encourage the parents to read aloud or tell a story to the child | 133 |
| Encourage the parents to sing for or with the child | 84 |
| Play music according to the child’s preferences. *Consult parents* | 83 |
| **Strategies to reduce noise** |  |
| Avoid loud talking in the child’s room. *Healthcare professionals should talk quietly* | 216 |
| Close the door if the staff is near to reduce noise | 209 |
| Use signs on patient’s door to communicate that the child is sleeping or it is nap time | 193 |
| Provide colleagues with noise feedback | 126 |
| Silence beepers and telephones if possible | 107 |
| In multiple-bed settings provide the child with headphones when listening to music or watching television | 94 |
| **Strategies to dim light** |  |
| Dim light by using curtains or blinds | 143 |
| Dim or turn off the artificial light around the child | 140 |
| Dim monitor screen light and turn away from child | 88 |
| Turn off tablets and smartphones before sleeping time | 79 |
| **Support physical activity** |  |
| **Strategies for increasing mobilization** |  |
| Document and evaluate daily mobilization goals | 294 |
| Provide physical therapy when appropriate | 280 |
| Incorporate physical therapy in the activities of the day | 259 |
| Encourage parent involvement in mobilization activities | 229 |
| Document restrictions for mobilization | 228 |
| Make activity goals visible in the child’s room | 219 |
| Encourage the child to be involved in activities of the day. *E.g. encourage the child to participate in personal hygiene* | 188 |
| Facilitate mobilization by removing or temporarily disconnect tubes and lines | 165 |
| Document needs for assistive devices. *Crutches, braces, wheelchair* | 163 |

^*^The total score was calculated from the experts’ overall ranking of the importance of each intervention as the number of experts who rated the specific intervention on rank 1 multiplied by the total number of interventions within the specific strategy. The number of experts who rated the intervention on rank 2 was multiplied by the number of interventions at the specific strategy minus 1 and so on. The sum of all rank placements was eventually summed for the intervention.

# Table F: Ranking of the interventions for children from 3 to 5 years of age

| Risk factor  Ranking interventions for **children from 3 to 5 years of age** | Total score^*^ |
| --- | --- |
| **Support cognition** |  |
| **Strategies for orientation provided by the healthcare professionals** |  |
| Speak calmly and clearly | 431 |
| Address child by name | 423 |
| Explain who you are when you approach the child. *Tell your name to the child* | 418 |
| Explain your role. *What are you going to do together with the child in your shift?* | 339 |
| Ensure that the child is provided with age-appropriate information | 256 |
| Stimulate the child’s orientation by asking or describing time and place. *Tell which weekday and date it is and where the child is.* | 246 |
| Make a fitting story with the child’s own words about the ward, hospital, and reason for admission so that everybody uses the same language that the family does. Keep the story in the present | 240 |
| Encourage consistent approach from staff caring for the child and consistent family support bedside for the child. | 229 |
| Explain plans for the day/evening/night*. E.g.* *should there be any examinations during the shift?* | 194 |
| Use a board to write the names of staff on shift who will care for the child and a little information about the child – what he likes to do and his interests | 121 |
| Staff should keep identification badge visible | 73 |
| **Strategies to promote a structure for the day** |  |
| Develop a day structure in collaboration with parents *What is the child’s normal day structure? When does the child goes to sleep? When does the child wake up in the morning?* | 74 |
| Provide bright light during the daytime*. Open blinds at daytime – no bright light at nap times* | 61 |
| **Strategies for improving the child’s environment** |  |
| Provide appropriate lighting according to the time of day | 118 |
| Provide single room for each child | 87 |
| Provide a clock and calendar within the child’s field of vision | 65 |
| **Strategies for improving visual or hearing impairment** |  |
| Ensure that the child uses eyeglasses if appropriate whenever awake and ensure that the glasses are clean. *Ask the parents when the child uses their eyeglasses during the daytime.* | 79 |
| Ensure that the child uses a hearing aid if appropriate when awake and ensure batteries are working | 56 |
| **Strategies to increase presence of parents** |  |
| Encourage the parents to be present | 153 |
| Encourage the parents to take part in the daily activities | 127 |
| Provide parents with oral information explaining the importance of being present and involved without making them feel blamed if they are not able to be present 24/7 | 110 |
| Encourage visits from grandparents and friends if appropriate | 60 |
| **Strategies to improve the hominess of the child’s surroundings** |  |
| Encourage presence of familiar objects around the bed. C*hild’s own pillow, pictures of family, friends, pets, posters, drawings.* | 125 |
| Encourage the child to do activities that they liked to do at home (e.g., watch television, use iPad). Consult parents | 91 |
| Encourage the child to listen to preferred music. *Consult parents* | 54 |
| **Support sleep** |  |
| **Strategies for bundling necessary nursing activities** |  |
| Schedule time for sleep. *Ask the parents about the usual sleep rhythm* | 197 |
| Bundle necessary nursing activities. *Conduct suctioning or diaper change when the child is awake if possible* | 152 |
| Adjust the default hours for medication administration | 116 |
| Adjust default hours for blood draws. *Do routine blood draws when the child is awake* | 112 |
| Adjust time for vital signs measurement. *Do routine measurement when the child is awake* | 98 |
| **Strategies to promote homelike sleep rituals** |  |
| Provide sleep objects such as teddy bear, sleeping pillow or cuddle cloth | 155 |
| Encourage the parents to read aloud or tell a story to the child | 139 |
| Play music according to the child’s preferences. *Consult parents* | 85 |
| Encourage the parents to sing for or with the child | 71 |
| **Strategies to reduce noise** |  |
| Avoid loud talking in the child’s room. *Healthcare professionals should talk quietly* | 217 |
| Close the door if the staff is near to reduce noise | 215 |
| Use signs on patient’s door to communicate that the child is sleeping or it is nap time | 199 |
| Provide colleagues with noise feedback | 120 |
| In multiple bed settings provide the child with headphones when listening to music or watching television | 98 |
| Silence beepers and telephones if possible | 96 |
| **Strategies to dim light** |  |
| Dim or turn off the artificial light around the child | 143 |
| Dim light by using curtains or blinds | 140 |
| Dim monitor screen light and turn away from child | 90 |
| Turn off tablets and smartphones before sleeping time | 77 |
| **Support physical activity** |  |
| **Strategies for increasing mobilization** |  |
| Document and evaluate daily mobilization goals | 273 |
| Provide physical therapy when appropriate | 250 |
| Incorporate physical therapy in the activities of the day | 241 |
| Encourage the child to be involved in activities of the day. *E.g. encourage the child to participate in personal hygiene* | 240 |
| Make activity goals visible in the child’s room | 237 |
| Encourage parent involvement in mobilization activities | 235 |
| Document restrictions for mobilization | 222 |
| Facilitate mobilization by removing or temporarily disconnect tubes and lines | 182 |
| Document needs for assistive devices. *Crutches, braces, wheelchair* | 145 |

^*^The total score was calculated from the experts’ overall ranking of the importance of each intervention as the number of experts who rated the specific intervention on rank 1 multiplied by the total number of interventions within the specific strategy. The number of experts who rated the intervention on rank 2 was multiplied by the number of interventions at the specific strategy minus 1 and so on. The sum of all rank placements was eventually summed for the intervention.

# Table G: Ranking of the interventions for children from 6 to 18 years of age

| Risk factor  Ranking interventions for **children from 6 to 18 years of age** | Total score^*^ |
| --- | --- |
| **Support cognition** |  |
| **Strategies for orientation provided by the healthcare professionals** |  |
| Explain who you are when you approach the child. *Tell your name to the child.* | 434 |
| Address child by name | 431 |
| Speak calmly and clearly | 408 |
| Explain your role. *What are you going to do together with the child in your shift?* | 357 |
| Stimulate the child’s orientation by asking or describing time and place. *Tell which weekday and date it is and where the child is* | 256 |
| Ensure that the child is provided with age-appropriate information | 250 |
| Make a fitting story with the child’s own words about the ward, hospital, and reason for admission so that everybody uses the same language that the family does. Keep the story in the present | 197 |
| Explain plans for the day/evening/night*. E.g.* *should there be any examinations during the shift?* | 221 |
| Encourage consistent approach from staff caring for the child and consistent family support bedside for the child. | 186 |
| Use a board to write the names of staff on the shift who will care for the child and a little information about the child – what he likes to do and his interests | 136 |
| Staff should keep identification badge visible | 94 |
| **Strategies to promote a structure for the day** |  |
| Develop a day structure in collaboration with parents *What is the child’s normal day structure? When does the child goes to sleep? When does the child wake up in the morning?* | 72 |
| Provide bright light during the daytime. Open blinds at daytime – no bright light at nap times. | 63 |
| **Strategies for improving the child’s environment** |  |
| Provide appropriate lighting according to the time of day | 95 |
| Provide a clock and calendar within the child’s field of vision | 92 |
| Provide single room for each child | 83 |
| **Strategies for improving visual or hearing impairment** |  |
| Ensure that the child uses eyeglasses if appropriate whenever awake and ensure that the glasses are clean. *Ask the parents when the child uses their eyeglasses during the daytime.* | 81 |
| Ensure that the child uses a hearing aid if appropriate when awake and ensure batteries are working | 54 |
| **Strategies to increase presence of parents** |  |
| Encourage the parents to be present | 151 |
| Encourage the parents to take part in the daily activities | 123 |
| Provide parents with oral information explaining the importance of being present and involved without making them feel blamed if they are not able to be present 24/7 | 100 |
| Encourage visits from grandparents and friends if appropriate | 76 |
| **Strategies to improve the hominess of the child’s surroundings** |  |
| Encourage the child to do activities that they liked to do at home (e.g., watch television, use iPad). Consult parents | 105 |
| Encourage presence of familiar objects around the bed. C*hild’s own pillow, pictures of family, friends, pets, posters, drawings.* | 92 |
| Encourage the child to listen to preferred music. *Consult parents* | 73 |
| **Support sleep** |  |
| **Strategies for bundling necessary nursing activities** |  |
| Schedule time for sleep. *Ask the parents about the usual sleep rhythm* | 201 |
| Bundle necessary nursing activities. *Conduct suctioning or diaper change when the child is awake if possible* | 151 |
| Adjust the default hours for medication administration | 109 |
| Adjust time for vital signs measurement. *Do routine measurement when the child is awake* | 109 |
| Adjust the default hours for blood draws. *Do routine blood draws when the child is awake* | 105 |
| **Strategies to promote homelike sleep rituals** |  |
| Play music according to the child’s preferences. *Consult parents* | 147 |
| Encourage the parents to read aloud or tell a story to the child | 121 |
| Provide sleep-objects such as teddy bear, sleeping pillow or cuddle cloth | 117 |
| Encourage the parents to sing for or with the child | 65 |
| **Strategies to reduce noise** |  |
| Close the door if the staff is near to reduce noise | 229 |
| Avoid loud talking in the child’s room. *Healthcare professionals should talk quietly* | 203 |
| Use signs on patient’s door to communicate that the child is sleeping or it is nap time | 184 |
| Provide colleagues with noise feedback | 118 |
| In multiple-bed settings provide the child with headphones when listening to music or watching television | 121 |
| Silence beepers and telephones if possible | 90 |
| **Strategies to dim light** |  |
| Dim or turn off the artificial light around the child | 138 |
| Dim light by using curtains or blinds | 135 |
| Turn off tablets and smartphones before sleeping time | 94 |
| Dim monitor screen light and turn away from child | 83 |
| **Support physical activity** |  |
| **Strategies for increasing mobilization** |  |
| Make activity goals visible in the child’s room | 284 |
| Encourage the child to be involved in activities of the day. *E.g. encourage the child to participate in personal hygiene* | 272 |
| Provide physical therapy when appropriate | 262 |
| Document and evaluate daily mobilization goals | 259 |
| Incorporate physical therapy in the activities of the day | 243 |
| Document restrictions for mobilization | 217 |
| Encourage parent involvement in mobilization activities | 199 |
| Facilitate mobilization by removing or temporarily disconnect tubes and lines | 154 |
| Document needs for assistive devices. *Crutches, braces, wheelchair* | 135 |

^*^The total score was calculated from the experts’ overall ranking of the importance of each intervention as the number of experts who rated the specific intervention on rank 1 multiplied by the total number of interventions within the specific strategy. The number of experts who rated the intervention on rank 2 was multiplied by the number of interventions at the specific strategy minus 1 and so on. The sum of all rank placements was eventually summed for the intervention.

# Table H: Non-pharmacologic Delirium management Program (NDB-PICU) – checklist for children from 0 to 2 years of age

| Non-pharmacologic program – (NDB-PICU) checklist  **Children from 0 to 2 years of age** | | Date:  Patient label: | | |
| --- | --- | --- | --- | --- |
|  | Tick off when completed | Day | Evening | Night |
| **Support cognition** | Speak calmly and clearly. | □ | □ | □ |
|  | Develop a day structure in collaboration with parents. | □ | □ | □ |
|  | Provide appropriate lighting according to the time of day. | □ | □ | □ |
|  | Ensure that the child uses eyeglasses and hearing aids if appropriate when awake, and ensure that the glasses are clean, and batteries are working. | □ | □ | □ |
|  | Encourage the parents to be present. | □ | □ | □ |
|  | Encourage presence of familiar objects around the bed. | □ | □ | □ |
| **Support sleep** | Schedule time for sleep  *Ask the parents about the usual sleep rhythm* | □ | □ | □ |
|  | Provide sleep objects from home such as teddy bear, sleeping pillow, or cuddle cloth. | □ | □ | □ |
|  | Avoid loud talking in the child’s room. | □ | □ | □ |
|  | Dim light by using curtains or blinds. | □ | □ | □ |
| **Support physical activity** | Document and evaluate daily mobilization goals. | □ | □ | □ |

# Table I: Non-pharmacologic Delirium management Program (NDB-PICU) – checklist for children from 3 to 5 years of age

| Non-pharmacologic program – (NDB-PICU) checklist  **Children from 3 to 5 years of age** | | Date:  Patient label: | | |
| --- | --- | --- | --- | --- |
|  | Tick off when completed | Day | Evening | Night |
| **Support cognition** | Explain who you are when you approach the child. *Tell your name to the child* | □ | □ | □ |
|  | Develop a day structure in collaboration with parents. | □ | □ | □ |
|  | Provide appropriate lighting according to the time of day. | □ | □ | □ |
|  | Ensure that the child uses eyeglasses and hearing aids if appropriate when awake, and ensure that the glasses are clean, and batteries are working. | □ | □ | □ |
|  | Encourage the parents to be present. | □ | □ | □ |
|  | Encourage presence of familiar objects around the bed. | □ | □ | □ |
| **Support sleep** | Schedule time for sleep.  *Ask the parents about the usual sleep rhythm* | □ | □ | □ |
|  | Provide sleep objects from home such as teddy bear, sleeping pillow, or cuddle cloth. | □ | □ | □ |
|  | Avoid loud talking in the child’s room. | □ | □ | □ |
|  | Dim or turn off the artificial light around the child. | □ | □ | □ |
| **Support physical activity** | Document and evaluate daily mobilization goals. | □ | □ | □ |

# Table J: Non-pharmacologic Delirium management Program (NDB-PICU) – checklist for children from 6 to 18 years of age

| Non-pharmacologic program – (NDB-PICU) checklist  **Children from 6 to 18 years of age** | | Date:  Patient label: | | |
| --- | --- | --- | --- | --- |
|  | Tick off when completed | Day | Evening | Night |
| **Support cognition** | Explain who you are when you approach the child. *Tell your name to the child* | □ | □ | □ |
|  | Develop a day structure in collaboration with parents. | □ | □ | □ |
|  | Provide appropriate lighting according to the time of day. | □ | □ | □ |
|  | Ensure that the child uses eyeglasses and hearing aids if appropriate when awake, and ensure that the glasses are clean, and batteries are working. | □ | □ | □ |
|  | Encourage the parents to be present. | □ | □ | □ |
|  | Encourage the child to do activities that they liked to do at home, e.g., watch television, use iPad. | □ | □ | □ |
| **Support sleep** | Schedule time for sleep.  *Ask the parents about the usual sleep rhythm* | □ | □ | □ |
|  | Play music according to the child’s preferences. *Consult parents* | □ | □ | □ |
|  | Close the door if the staff is near to reduce noise. | □ | □ | □ |
|  | Dim or turn off the artificial light around the child. | □ | □ | □ |
| **Support physical activity** | Make activity goals visible in the child’s room. | □ | □ | □ |
